# NOD1 Agonist Protects Against Lipopolysaccharide and D-Galactosamine-Induced Fatal Hepatitis Through the Upregulation of A20 Expression in Hepatocytes

**DOI:** 10.3389/fimmu.2021.603192

**Published:** 2021-03-04

**Authors:** Fang Jia, Fuxue Deng, Pan Xu, Shiying Li, Xuefu Wang, Peng Hu, Hong Ren, Shiwen Tong, Wenwei Yin

**Affiliations:** ^1^Key Laboratory of Molecular Biology for Infectious Diseases (Ministry of Education), Department of Infectious Diseases, Institute for Viral Hepatitis, The Second Affiliated Hospital of Chongqing Medical University, Chongqing, China; ^2^Department of Endocrinology, The Second Affiliated Hospital of Xi'an Jiaotong University (Xibei Hospital), Xi'an Jiaotong University, Xi'an, China; ^3^Department of Cardiovascular Medicine, The Second Affiliated Hospital of Xi'an Jiaotong University (Xibei Hospital), Xi'an Jiaotong University, Xi'an, China; ^4^School of Pharmacy, Anhui Medical University, Hefei, China; ^5^Department of Clinical Nutrition, The Second Affiliated Hospital of Chongqing Medical University, Chongqing, China

**Keywords:** NOD1, A20, acute liver failure, LPS/D-GalN, apoptosis

## Abstract

Increasing evidence suggests that NODs are involved in liver diseases; however, the underlying mechanisms remain obscure. In the present study, we analyzed the effect of NOD1 agonist pretreatment on acute liver failure induced by lipopolysaccharide (LPS) in D-galactosamine (D-GalN)-sensitized mice. We found that pretreatment with the NOD1 agonist markedly reduced LPS/D-GalN-induced mortality, elevation of serum ALT levels, and hepatocyte apoptosis. The protective effect of NOD1 agonist was independent of tumor necrosis factor (TNF)-α inhibition. NOD1 agonist pretreatment also attenuated TNF-α/D-GalN-induced apoptotic liver damage. The anti-apoptotic protein A20 expression was more pronounced in NOD1 agonist pretreated mice than in controls, and knockdown of A20 abrogated the protective effect of NOD1 agonist on LPS/D-GalN-induced liver injury and hepatocyte apoptosis. Further experiments showed that NOD1 agonist-induced A20 upregulation required the presence of kupffer cells and TNF-α. Taken together, our data strongly indicate that NOD1 is involved in the regulation of liver injury and could be a potential therapeutic target for liver diseases.

## Introduction

Acute liver failure (ALF), caused by massive destruction of hepatocytes with resultant severe impairment of liver function, is a serious problem in clinical practice (1). Apoptosis of hepatocytes is a seminal feature of ALF (2). Hepatotoxic substances such as ethanol, acetaminophen and cytostatic drugs can induce hepatocyte apoptosis (3). Apoptosis of hepatocytes is also a feature of viral hepatitis, sepsis, ischemic liver injury, and cholestasis (3). Hepatocyte apoptosis and necrosis, when massive, result in ALF (4). Identifying and increasing the anti-apoptotic armamentarium of hepatocytes is important to develop effective interventions to prevent or treat ALF.

The LPS/D-GalN model has been widely used to explore the mechanisms of ALF and screen for potential hepatoprotective drugs (5, 6). In this model, LPS binds to and activates kupffer cells, resulting in a liberation of large amounts of proinflammatory cytokines, such as TNF-α, IL-1, and IL-6 (7). Among them, TNF-α is a terminal mediator for both hepatocyte apoptosis and organ failure (8). TNF-α-induced hepatocyte apoptosis has been identified as an early and possibly causal event in LPS/D-GalN-induced acute liver failure (8–10). Massive hepatocyte apoptosis induced by TNF-α was shown to be the central mechanism of liver damage in this model (11). D-GalN, which induces a selective transcriptional block in hepatocytes, amplifies the toxicity of LPS and TNF-α to liver without damaging other organs or tissues (12).

Nucleotide oligomerization domain receptor 1 (NOD1) is a cytoplasmic pattern recognition receptor that recognizes bacterial cell wall components and has been associated with many inflammatory diseases in humans, highlighting their significant immunologic role (13). NOD1 is widely expressed in liver parenchymal and non-parenchymal cells and the liver is constantly exposed to gut-derived bacterial products, suggesting that NOD1 might be involved in liver diseases (14). For example, ischemia-reperfusion and CCL4-induced hepatic inflammation and hepatocellular damage are greatly attenuated in NOD1-deficient mice (15). NOD1 agonist application in the liver could activate T cells and suppresses hepatitis B virus replication (16). Stimulation of hepatocytes with NOD1 ligand increased CC and CXC chemokine release and nitric oxide (NO) production (17).

In the present study, we sought to determine the effect of NOD1 activation by its agonist on ALF induced by LPS/D-GalN. Our data show that NOD1 agonist treatment prevented LPS/D-GalN-induced fatal hepatitis through the upregulation of A20 expression in hepatocytes.

## Materials and Methods

### Reagents

LPS (*Escherichia coli*, O55:B5) and D-GalN were purchased from Sigma-Aldrich (St. Louis, MO, USA). Two NOD1 agonists, C14-Tri-LAN-Gly, and C12-iE-DAP, were obtained from InvivoGen (Toulous, France). Recombinant mouse TNF-α was purchased from Biolegend (San Diego, USA). Clodronate-liposomes was purchased from FormuMax (California, USA).

### Animal and Treatments

Male C57BL/6J mice (6–8 week-old) were provided by the Institute of Laboratory Animal Sciences, Chongqing Medical University (Chongqing, China), while TNF-α KO mice in C57BL/6J background were provided by Xuemei Zhang (College of Laboratory Medicine, Chongqing Medical University). Mice were housed under a 12-h light-dark cycle in a barrier housing facility. The house facility complies with the national standard of Laboratory Animal-Requirements of Environment and Housing Facilities (GB 14925-2010), and the facility permit number is SYXK 2018-003. The animals were treated according to the guidelines of the Institutional Animal Care and Use Committee of Chongqing Medical University. Ethical approval was obtained from the Ethics Committee of the The Second Affiliated Hospital of Chongqing Medical University.

Two mouse models of LPS/D-GalN and TNF-α/D-GalN were used to induce liver injury. For LPS-induced liver injury, mice were co-injected i.p., with LPS (7.5 μg/kg) and D-GalN (500 mg/kg). In the TNF-α/D-GalN model, 0.4 μg/mouse TNF-α and 750 mg/kg D-GalN were co-injected i.p., into mice. C14-Tri-LAN-Gly was administered i.p., at 5 μg/mouse at 6 h before LPS/D-GalN or TNF-α/D-GalN co-injection. ShRNA was purchased from Genechem (Shanghai, China). The target sequences for shRNA design were CAAAGCACTTATTGACAGA for A20 and TTCTCCGAACGTGTCACGT for GFP (shControl). Mice were hydrodynamically injected with shRNA (30 μg/mouse) at 72 h before C14-Tri-LAN-Gly challenge. For Kupffer cell depletion, mice were injected with clodronate-liposomes i.v., containing 200 and 100 μl/mouse at 48 and 24 h, respectively, before C14-Tri-LAN-Gly challenge. The vehicle control groups received the same volume PBS.

### Transminase Assay

To assay for serum ALT levels, we slightly anesthetized the mice with ether and obtained the blood sample from the eyepit. Serum was mixed with ALT assay solution (Nanjing JianCheng, Nanjing, China) and analyzed using a spectrophotometer following the supplier's protocol.

### Measurement of Cytokine Levels

The serum samples were kept at −20°C until ready for cytokine measurement. Liver tissues homogenates were prepared following the protocol of the ELISA kits. The levels of TNF-α and IL-6 were measured using commercially available ELISA kits from DAKEWE (Beijing, China).

### Histology and TUNEL Assay

Liver samples were excised and immediately fixed within 10% neutral-buffered formalin. Samples were embedded in paraffin and cut into 5 μm sections. Livers sections were affixed to slides, deparaffinized, and stained with hematoxylin and eosin (H&E) to determine morphologic changes. Apoptotic hepatocytes were detected with the terminal deoxynucleotidyl transferase-mediated nick end labeling (TUNEL) assay by using the *in situ* Cell Death Detection Kit, POD (Roche, Mannheim, Germmany).

### Western Blot

Whole-cell protein and liver tissue homogenates were extracted for Western blot analysis. Proteins were desaturated with loading buffer and boiled for 5 min. Exactly 50 μg of total protein was loaded in SDS-PAGE. After electrophoresis, proteins were transferred onto PVDF membrane, and blotted against cleaved caspase-3, A20, and GAPDH (Cell Signaling Technology, Beverly, USA) primary antibodies overnight at 4°C. Membranes were washed with 0.05% Tween-20 in TBS and incubated with a 1:10000 dilution horseradish peroxidase-conjugated secondary antibodies, and chemiluminescence was performed according to the manufacturer's protocol (Bio-Rad, USA).

### Real-Time PCR

Total RNA was extracted from liver tissue and cells by using TRIzol reagent (Life Technology, Carlsbad, USA). Cellular RNA (1 μg) was reverse-transcribed into cDNA by using the PrimeScriptTM RT reagent kit with gDNA Eraser (Takara, Japan). Quantitative PCR was performed using a sequence detector (Bio-Rad, USA) and a SYBR Premix Ex Taq (Takara, Japan), according to the manufacturer's instructions. The primer sequences used were as follows: GAPDH, CATCACTGCCACCCAGAAGACTG (forward) and ATGCCAGTGAGCTTCCCGTTCAG (reverse); A20, AGCAAGTGCAGGAAAGCTGGCT (forward) and GCTTTCGCAGAGGCAGTAACAG (reverse); c-IAP1, GATACGGATGAAGGGTCAGGAG (forward) and GGGTCAGCATTTTCTTCTCCTGG XIAP, GGCAGAATATGAAGCACGGATCG (forward) and CACTTGGCTTCCAATCCGTGAG (reverse); and Flip, GCTCTACAGAGTGAGGCGGTTT (forward) and CACCAATCTCCATCAGCAGGAC (reverse). The relative mRNA levels of specific genes were normalized to those of GAPDH. Gene expression values were then calculated using the ΔΔCt method, as previously described (18).

### Liver Mononuclear Cells and Primary Hepatocyte Isolation

Liver mononuclear cells (MNCs) were isolated, as described previously (19). The liver sample was passed through a 200-gauge stainless steel mesh. The cells were separated with 40 and 70% Percoll (GE Healthcare Pharmacia, USA) by centrifugation at 750 ×g for 15 min. Liver MNCs were collected from the interphase.

Primary hepatocytes were isolated using the collagenase perfusion method, as described previously (20). The liver was perfused with two kinds of solution sequentially. Solution I was composed of KCl, NaCl, Na_2_HPO_4_, EGTA, and Tricine. Solution B was composed of DMEM and 0.075% collagenase I (Biosharp, China). Hepatocytes were then separated with 40% Percoll by centrifugation at 400 ×g for 10 min. The isolated primary hepatocytes were cultured in Dulbecco's Modified Eagle's Medium supplemented with 10% fetal bovine serum overnight prior to treatments.

### Flow Cytometric Analysis

The cell surface expression of TLR4 and TNFR1 was assessed using FITC-conjugated anti-mouse TLR4 (eBioscience, San Diego, USA) and PE-conjugated anti-mouse TNFR1 (Biolegend, San Diego, USA). After blocking with anti-FcrR, cells were incubated with saturation mounts of indicated fluorescence-labeled mAbs at 4°C for 30 min in darkness and then washed twice with PBS. Date were recorded from the stained cells using a FACSCanto II flow cytometer (BD Biosciences, USA) and analyzed using the FlowJo software.

### Statistical Analysis

Date were expressed as the mean ± SEM. The results were analyzed using log-rank test (for survival) and Mann-Whitney test. *P*-values <0.05 were considered as statistically significant.

## Results

### NOD1 Agonist Pretreatment Prevented LPS/D-GalN-induced Fatal Hepatitis

**Figure 1:**
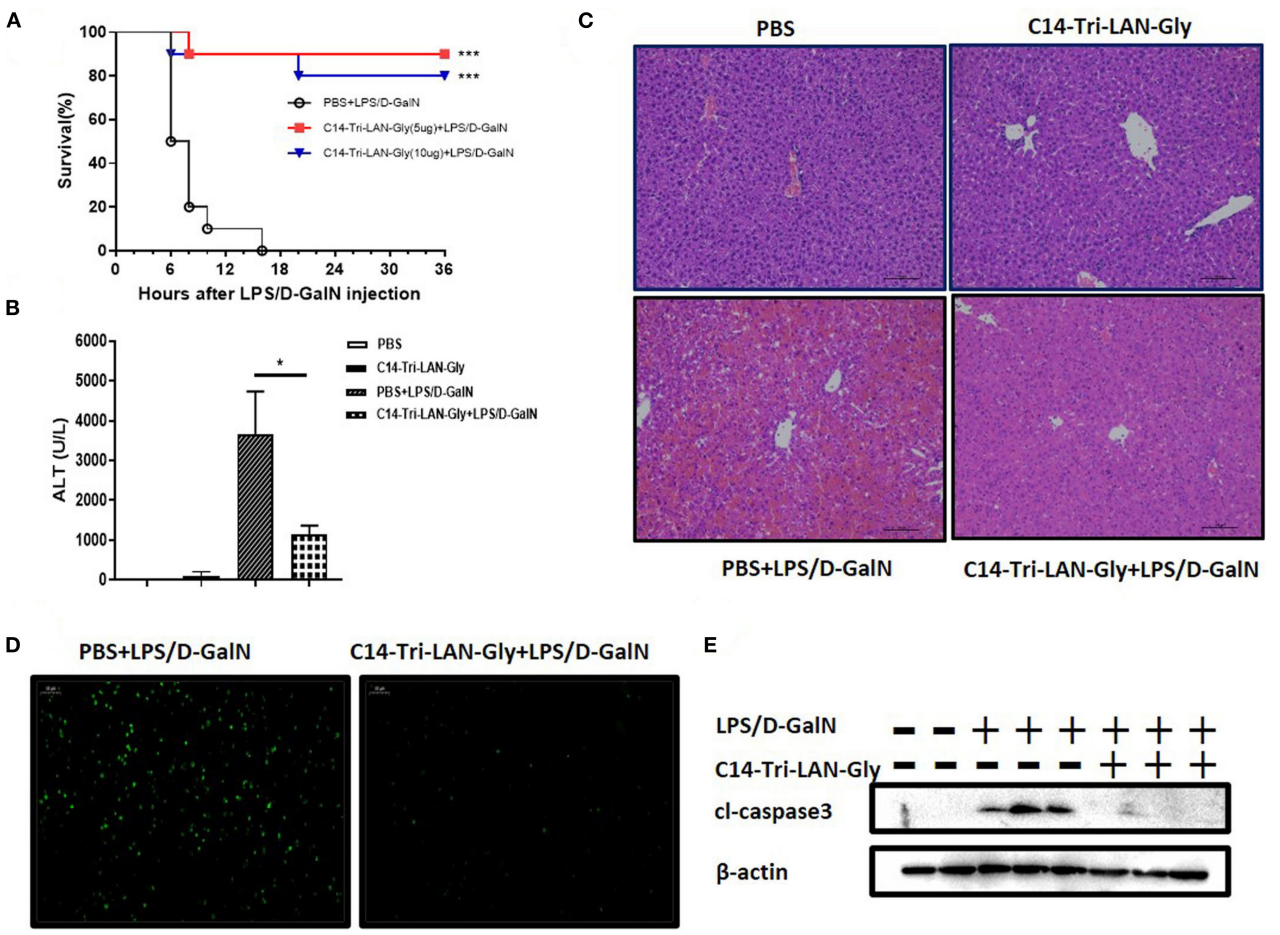
C14-Tri-ALN-Gly protects mice from LPS/D-GalN-induced liver injury. Mice were i.p., injected with C14-Tri-LAN-Gly 5 or 10 μg/mouse for 6 h. Subsequently, acute liver injury was induced by application of LPS (7.5 μg/kg)/D-GalN (500 mg/kg). Mice were injected with the same volume of PBS as the vehicle control. **(A)** Survival curves of LPS/D-GalN mice treated with C14-Tri-LAN-Gly (*n* = 10). Survival rates were analyzed using the log-rank test. **(B)** Serum levels of ALT were measured at 6 h after LPS/D-GalN injection. **(C,D)** Representative liver histopathology with H&E staining and apoptosis detection with the TUNEL assay (apoptotic cells are stained in green) at 6 h after LPS/D-GalN injection. Original magnification is ×200. **(E)** Protein expression for cleaved caspase-3 was detected by Western blot assay at 6 h after LPS/D-GalN challenge. Data are presented as mean ± SEM of three independent experiments (*n* = 3–5). *p < 0.05, ***p < 0.001.

Co-injection with LPS and D-GalN in mice induced severe liver injury with a high mortality in a short time. C14-Tri-LAN-Gly, a highly selective and potent NOD1 agonist, was used to activate NOD1. To investigate the effects of NOD1 agonist on LPS/D-GalN-induced liver injury, we treated the mice with C14-Tri-LAN-Gly (5 or 10 μg/mouse) 6 h before LPS/D-GalN challenge. As shown in Figure 1A, both dosage of C14-Tri-LAN-Gly pretreatment markedly increased the survival rates of LPS/D-GalN-treated mice. The protective effect of C14-Tri-LAN-Gly was also confirmed by results revealing that C14-Tri-LAN-Gly pretreatment inhibited elevation of serum ALT levels in LPS/D-GalN-treated mice (Figure 1B). Furthermore, histological analysis showed that the hepatic microarchitecture was destroyed and appeared to have massive hemorrhage at the site of the sinusoids in control mice, while C14-Tri-LAN-Gly-pretreated mice had an intact liver architecture with no signs of hemorrhage (Figure 1C).

TUNEL assay showed significant hepatocyte apoptosis in the livers of mice treated with LPS/D-GalN, which was markedly prevented by C14-Tri-LAN-Gly pretreatment (Figure 1D). Moreover, pretreatment with C14-Tri-LAN-Gly inhibited the expression of cleaved-caspase3, which is a major executioner of apoptosis and essential for DNA fragmentation, in the liver of LPS/D-GalN-treated mice (Figure 1E). In addition to C14-Tri-LAN-Gly, we have also studied the effect of another NOD1 agonist C12-iE-DAP, which is widely used, on LPS/D-GalN-induced hepatitis. Similarly, administration of C12-iE-DAP improved the survival rates of LPS/D-GalN-treated mice and suppressed LPS/D-GalN-induced elevation of ALT markedly (Supplementary Figure 1). Collectively, these results indicate that NOD1 agonist pretreatment protected mice from LPS/D-GalN-induced lethal hepatitis, probably by inhibiting hepatocyte apoptosis.

### The Protective Effect of NOD1 Agonist Was Not Attributed to impaired TNF-α Production

**Figure 2:**
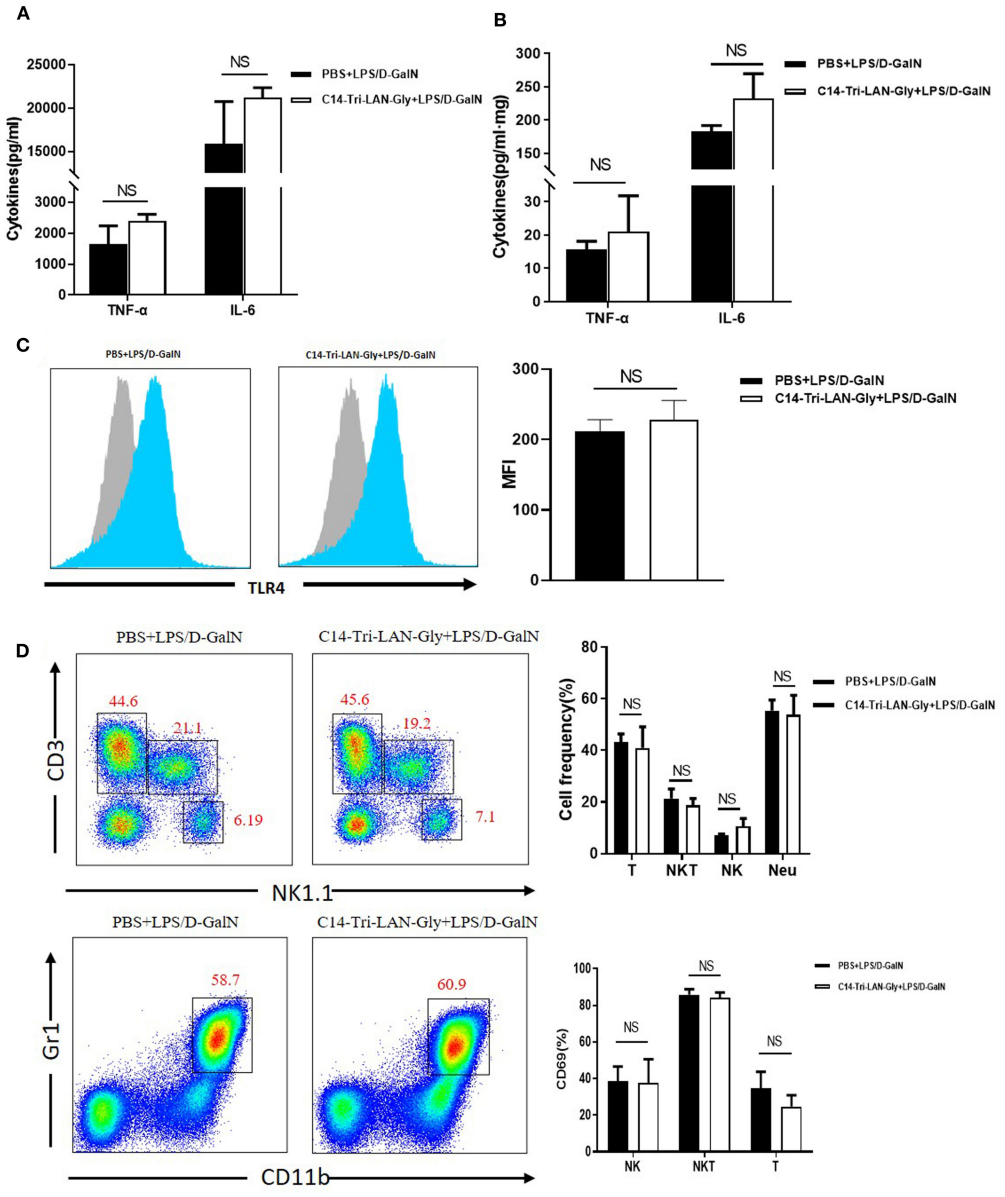
C14-Tri-LAN-Gly does not influence TNF-α production and TLR4 expression by Kupffer cells in LPS/D-GalN mouse model. **(A,B)** TNF-α and IL-6 levels in serum and liver homogenate 2 h after LPS/D-GalN challenge. (**C)** TLR4 expression on Kupffer cells from mice 2 h after LPS/D-GalN challenge was determined by flow cytometry. Kupffer cells were gated on the F4/80+ population. Blue histograms represent the PBS or C14-Tri-LAN-Gly treatment group, gray histograms represent the isotype. The right bar chart shows the mean fluorescence intensity (MFI) of the TLR4 expression. **(D)** Hepatic mononuclear cells from LPS/D-GalN-challenged mice were isolated and then marked with specific antibodies. The percentages of hepatic lymphocytes (NK, NKT, and T cells) and neutrophils, and lymphocyte activation (CD69 expression) were detected by flow cytometry. Data are presented as mean ± SEM of three independent experiments (*n* = 3–4).

Because TNF-α is a key mediator in LPS/D-GalN-induced acute liver injury (21), we determined whether C14-Tri-LAN-Gly pretreatment diminished TNF-α production. LPS/D-GalN injection led to a remarkable elevation in the serum and liver TNF-α and IL-6; surprisingly, pretreatment with C14-Tri-LAN-Gly did not affect their production (Figures 2A,B). Further analysis showed NOD1 agonist pretreatment did not diminish serum TNF-α levels at different time points (1, 2, 3, and 4h) after LPS/D-GalN administration (Supplementary Figure 2). These results suggest the protective effect of C14-Tri-LAN-Gly was independent of TNF-α inhibition.

Down-regulation of TLR4 has been suggested as a mechanism for LPS tolerance induction (22). However, flow cytometry analysis showed that C14-Tri-LAN-Gly did not inhibit the LPS-enhanced TLR4 expression on the surface of Kupffer cells (Figure 2C). Additionally, C14-Tri-LAN-Gly pretreatment also did not influence the percentages of hepatic lymphocytes (NK.NKT and T cells) and neutrophils, and lymphocyte activation (CD69 expression) (Figure 2D). Further, we examined the effect of C14-Tri-LAN-Gly treatment alone on hepatic leukocyte infiltration and observed that hepatic lymphocytes (NK.NKT and T cells) and neutrophils were not significantly changed after C14-Tri-LAN-Gly injection (Supplementary Figure 3).

### NOD1 Agonist Similarly Attenuated TNF-α/D-GalN-induced Liver Injury

**Figure 3:**
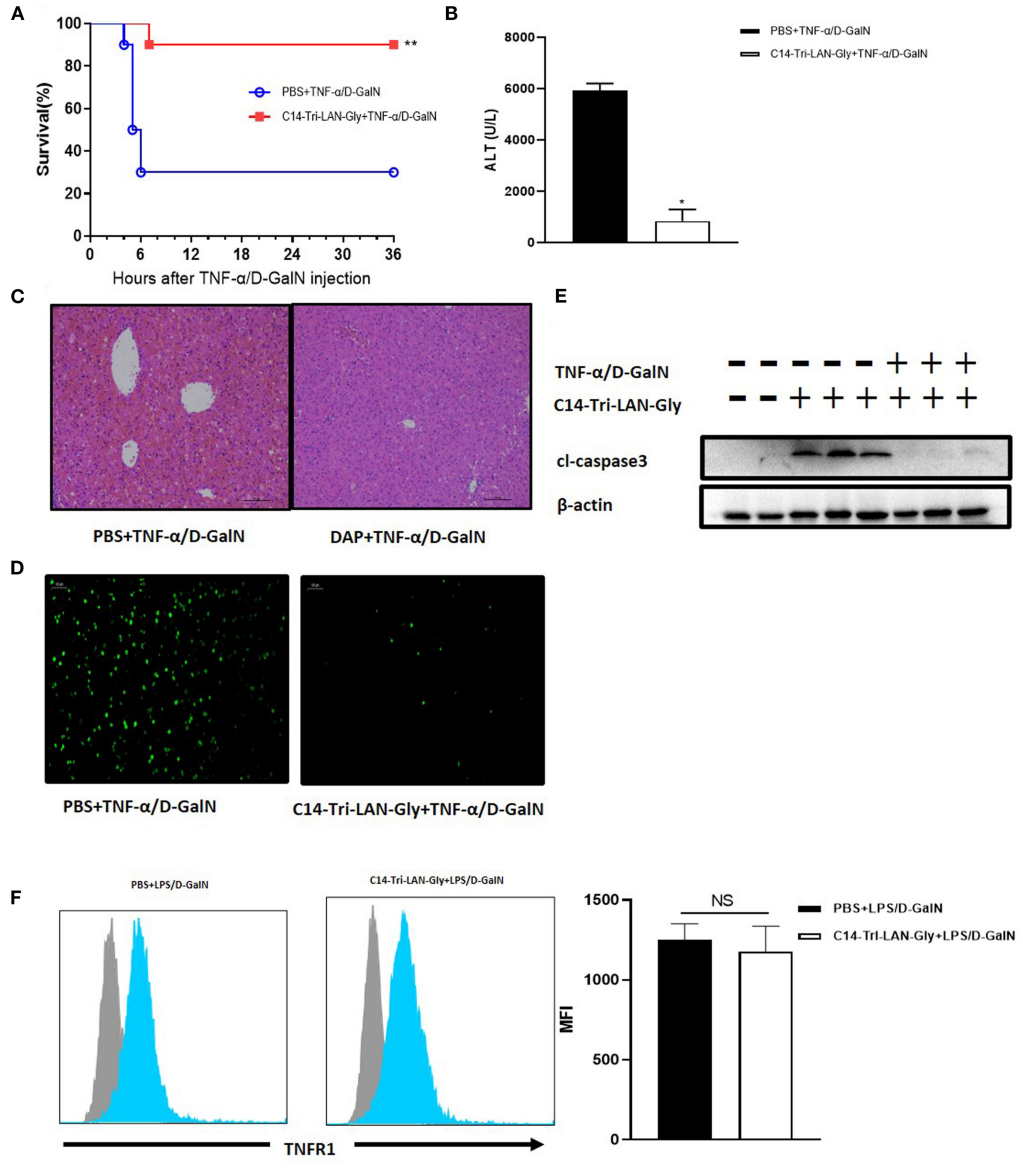
Protective effects of C14-Tri-ALN-Gly on the TNF-α/D-GalN mouse model. Mice were i.p., injected with C14-Tri-LAN-Gly for 6 h. Subsequently, acute liver injury was induced by application of TNF-α (0.4 μg/mouse)/D-GalN (500 mg/kg). **(A)** Survival curves of TNF-α/D-GalN mice treated with C14-Tri-LAN-Gly (*n* = 10). **(B)** Serum levels of ALT were measured 6 h after TNF-α/D-GalN injection. **(C,D)** Representative liver histopathology with H&E staining and apoptosis detection with the TUNEL assay (apoptotic cells are stained in green) at 6 h after TNF-α/D-GalN injection. Original magnification is 200×. **(E)** Protein expression for cleaved-caspase3 was detected by Western blot assay at 6 h after TNF-α/D-GalN challenge. **(F)** Primary hepatocytes were isolated 3 h after TNF-α/D-GalN co-injection, and flow cytometry was used to determine the TNFR1 expression levels on hepatocytes. Blue histograms represent the PBS or C14-Tri-LAN-Gly-treated group, while gray histograms represent the isotype. The right bar chart shows the mean fluorescence intensity (MFI) of TNFR1 expression. Data are presented as mean ± SEN of three independent experiments (*n* = 3–5). *p < 0.05, **p < 0.01.

Considering that the protective effect of C14-Tri-LAN-Gly on LPS/D-GalN induced liver injury is not attributable to decreased TNF-α production, we speculate that C14-Tri-LAN-Gly may influence TNF-α-induced hepatocyte apoptosis. To test this hypothesis, we investigated the effect of C14-Tri-LAN-Gly on TNF-α/D-GalN-induced liver failure. Similarly, C14-Tri-LAN-Gly pretreatment significantly increased the survival rate of TNF-α/D-GalN-treated mice (Figure 3A). The protective effect of C14-Tri-LAN-Gly was also confirmed by ALT assay and histological analysis (Figures 3B,C).

The TUNEL assay further revealed that C14-Tri-LAN-Gly reduced the frequency of apoptosis induced by TNF-α/D-GalN to a significant extent (Figure 3D). Western blot also showed that the expression of cleaved-caspase3 significantly decreased in C14-Tri-LAN-Gly-treated mice (Figure 3E). Together, these results indicate that C14-Tri-LAN-Gly protects mice from TNF-α/D-GalN-induced lethal hepatitis by inhibiting hepatocyte apoptosis.

TNF-α-induced apoptosis is mediated by the TNF receptor-1 (TNFR1) (23). To clarify whether C14-Tri-LAN-Gly exerts a protective effect by downregulating the amount of TNFR1 on hepatocytes, we isolated primary hepatocytes from C14-Tri-LAN-Gly-pretreated or control mice and assessed TNFR1 expression by flow cytometry. As shown in Figure 3F, C14-Tri-LAN-Gly pretreatment did not affect hepatocyte TNFR1 expression in LPS/D-GalN-treated mice. Thus, the protective effect of C14-Tri-LAN-Gly was not attributed to downregulation of TNFR1.

### NOD1 Agonist Further Upregulated the A20 Expression in the Liver of LPS/D-GalN-Challenged Mice

**Figure 4:**
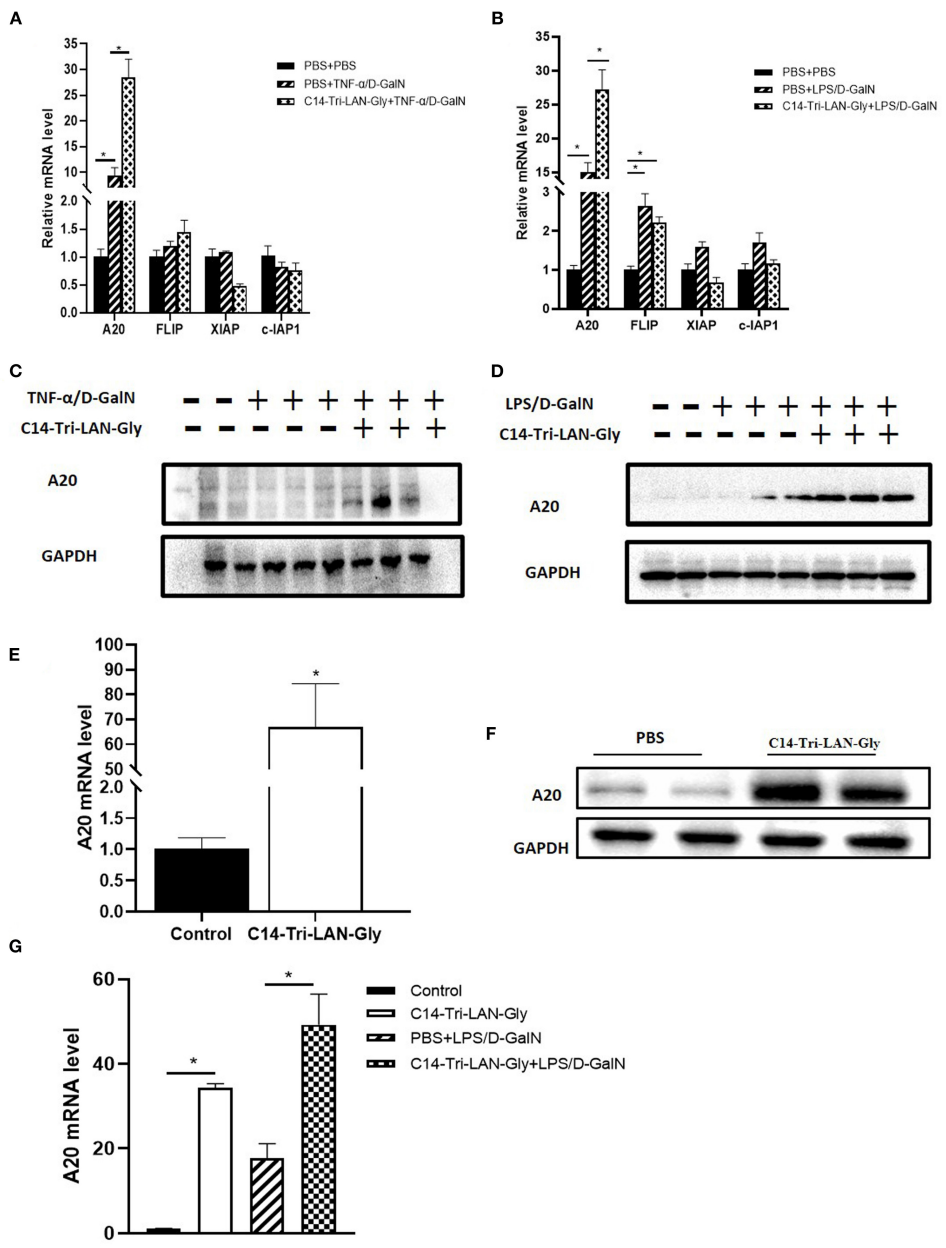
C14-Tri-LAN-Gly treatment upregulates A20 expression in liver. **(A,B)** mRNA levels of A20, c-Flip, Xiap, and c-IAP1 in liver after TNF-α/D-GalN or LPS/D-GalN challenge. **(C,D)** Total liver lysates were subjected to western blot analysis for the indicated proteins. **(E,F)** Mice were injected with C14-Tri-LAN-Gly or PBS alone, the A20 mRNA and protein expression levels in liver were detected. **(G)** Primary hepatocytes were isolated from the indicated mice and the mRNA levels of A20 were measured using RT-qPCR. Data are presented as mean ± SEM of three independent experiments (*n* = 3). ^*^p < 0.05.

To further clarify the mechanism by which C14-Tri-LAN-Gly protects hepatocytes from apoptosis, we determined the expression of some potential anti-apoptosis genes in livers of TNF-α/D-GalN or LPS/D-GalN mice with real-time PCR assay. Notably, the expression of A20 was markedly enhanced in the C14-Tri-LAN-Gly-pretreated mice compared with control mice (Figures 4A,B). In addition, western blot results confirmed that C14-Tri-LAN-Gly dramatically upregulated the expression of A20 protein in the liver of LPS/D-GalN mice (Figures 4C,D).

We also examined the effect of C14-Tri-LAN-Gly treatment alone on the expression of A20. As shown in Figures 4E,F, both mRNA level and protein level of A20 was significantly increased in the liver after C14-Tri-LAN-Gly challenge. Next, we isolated hepatocytes from mouse liver to evaluate A20 expression changes in hepatocytes. We found that A20 expression in hepatocytes was increased in LPS/D-GalN treated mice and C14-Tri-LAN-Gly further enhanced this effect (Figure 4G).

### Knockdown of A20 Abrogated the Protective Effect of NOD1 Agonist on LPS/D-GalN-induced Liver Injury

**Figure 5:**
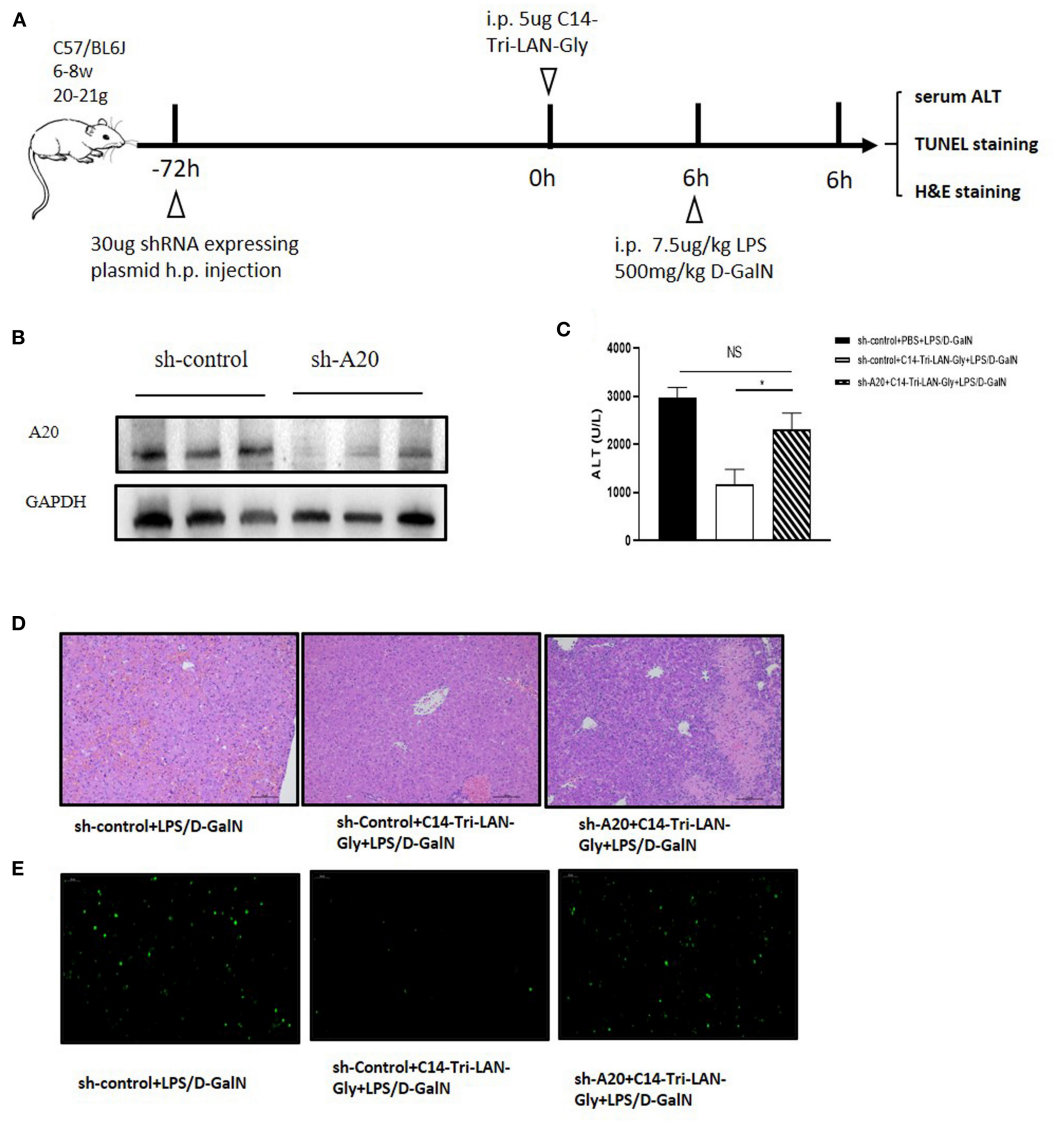
A20 knockdown abrogates the ability of C14-Tri-LAN-Gly to attenuate LPS/D-GalN-induced hepatitis. **(A)** Procedure for the injection of shRNA 72 h prior to C14-Tri-LAN-Gly administration. **(B)** The expression level of A20 in liver was determined by Western blot analysis. **(C)** Serum ALT, **(D)** liver tissue H&E staining, and **(E)** liver tissue TUNEL staining were determined 6 h after LPS/D-GalN coadministration (apoptotic cells are stained in green). Original magnification is ×200. Data are representative of mean ± SEM (*n* = 4) *p < 0.05.

To determine whether up-modulation of A20 expression was actually involved in the protective effect of C14-Tri-LAN-Gly on LPS/D-GalN-induced liver injury, we designed shRNA directed specifically against murine A20 (shA20). For hepatocyte-specific knockdown of A20 (24), mice were hydrodynamically injected with shA20 or control shRNA (shControl) (30 μg/mouse) at 72 h before C14-Tri-LAN-Gly or PBS challenge (Figure 5A). In comparison to shControl, shA20 down-modulated A20 expression *in vivo* by ~70% (Figure 5B). A20 knockdown in C14-Tri-LAN-Gly -pretreated mice restored their sensitivity toward LPS/D-GalN-induced liver damage as manifested by elevated serum ALT levels and histology (Figures 5C,D). Restoration of apoptosis by shA20 in C14-Tri-LAN-Gly-pretreated mice could also be observed in liver slices stained with TUNEL (Figure 5E). These experiments indicate that the upregulated A20 expression is responsible for the protective effect of C14-Tri-LAN-Gly on LPS/D-GalN-induced liver injury.

### NOD1 Agonist-induced A20 Upregulation Was Dependent on Kupffer Cell-derived TNF-α

**Figure 6:**
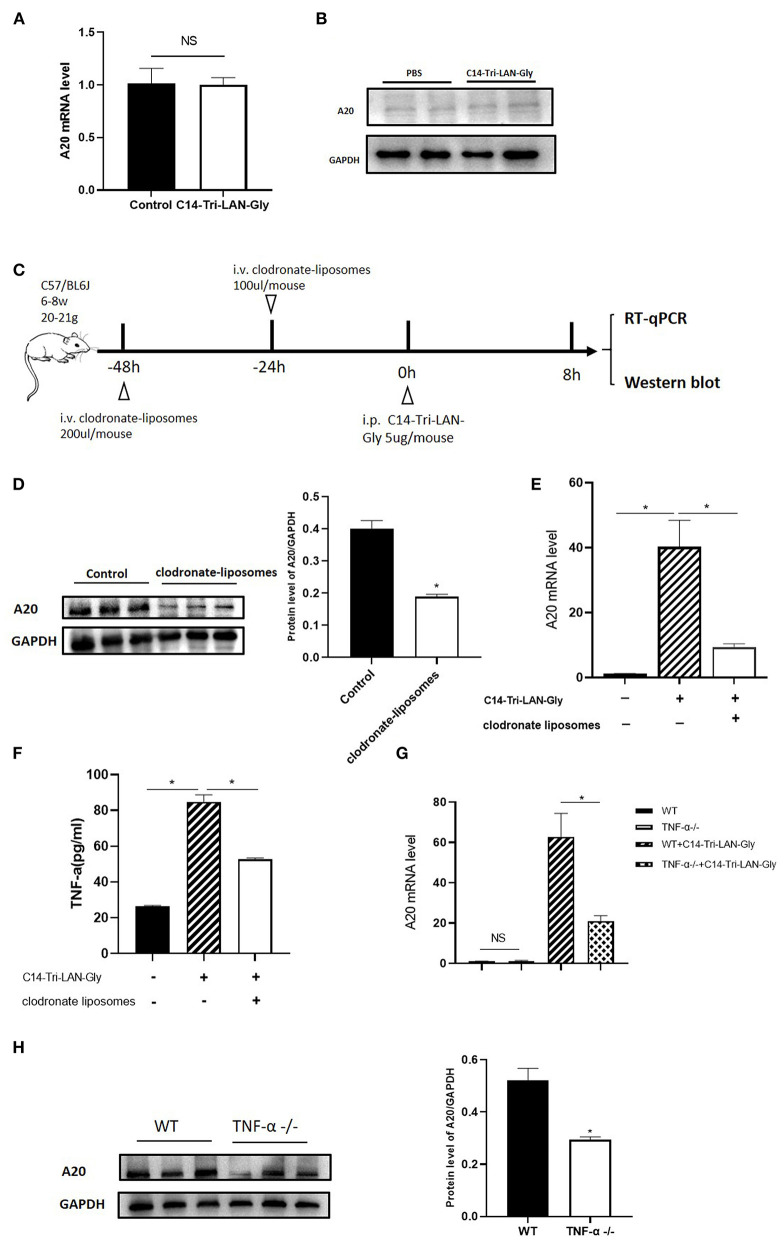
C14-Tri-LAN-Gly-induced A20 upregulation is dependent on Kupffer cell-derived TNF-α. **(A,B)** Primary hepatocytes were isolated and stimulated with C14-Tri-LAN-Gly *in vitro* for 30 min, and A20 expression was determined by real-time PCR and Western blot. **(C)** Procedure for the injection of clodronate-liposomes 48 and 24 h prior to C14-Tri-LAN-Gly administration, mice were sacrificed 8 h after C14-Tri-LAN-gly infusion. **(D,E)** A20 protein and mRNA expression levels in the liver were measured using Western blot analysis and RT-qPCR, respectively. **(F)** Serum levels of TNF-α induced by C14-Tri-LAN-Gly were determined by Elisa test. **(G,H)** TNF-α^−/−^ and wild type mice were treated with C14-Tri-LAN-Gly, and A20 expression was determined by real-time PCR and Western blot assay. Data are representative of mean ± SEM. (*n* = 3) *p < 0.05.

To further investigate the mechanism of NOD1 agonist-induced A20 upregulation, we isolated primary hepatocytes and treated them with C14-Tri-LAN-Gly, and total RNA and protein were extracted to analyze A20 expression. As shown in Figures 6A,B, real-time PCR and western blot showed that A20 expression was not markedly different between PBS and C14-Tri-LAN-Gly group. Therefore, C14-Tri-LAN-Gly may not directly induce A20 upregulation in hepatocytes. As kupffer cells express high levels of NOD1 (14), we investigated whether kupffer cells were involved in the induced upregulation of A20 by C14-Tri-LAN-Gly. Kupffer cells were depleted with clodronate-liposomes prior to C14-Tri-LAN-Gly treatment (Figure 6C). The C14-Tri-LAN-Gly-induced upregulation of A20 was markedly reduced in kupffer cell-depleted mice (Figures 6D,E), indicating that kupffer cells are indispensable in this process.

A20 was originally identified as a TNF-inducible gene (25). C14-Tri-LAN-Gly treatment resulted in an increase in serum TNF-α levels, which was abolished by depletion of kupffer cells (Figure 6F). To confirm the role of TNF-α in the upregulation of A20 by C14-Tri-LAN-Gly, we treated B6 and TNF-α^−/−^ (TNF-α knockout) mice with C14-Tri-LAN-Gly for 8 h, then the expression levels of A20 mRNA and protein were measured by real-time PCR and western blot assay. As shown in Figures 6G,H, both mRNA level and protein level of A20 was markedly reduced in TNF-α^−/−^ mice. These results indicate that TNF-α produced by kupffer cells induced A20 upregulation in hepatocytes following C14-Tri-LAN-Gly treatment.

## Discussion

Treatment of mice with LPS/D-GalN induces fulminant hepatitis, which is mediated by TNF-α and characterized by massive hepatic apoptosis. In this study, we defined a protective role of C14-Tri-LAN-Gly, a NOD1 agonist, in LPS/GalN-induced (TNF-α-mediated) hepatocyte killing. This protection was associated with the upregulation of A20 in hepatocytes.

The liver is a sentinel organ in a unique position to monitor pathogen-associated molecules in the portal and systemic circulations. NOD1 is highly expressed in liver and is stimulated mainly by bacterial cell wall components from gram-negative bacteria (26), which make up a large part of gut flora. Therefore, it is likely that NOD1 participates in the progression of liver disease. Here, we provide evidences that NOD1 activation attenuated liver injury by demonstrating that pretreatment with C14-Tri-LAN-Gly protected against LPS/D-GalN-induced lethal hepatitis.

In the LPS/D-GalN-induced liver injury model, neutralization of TNF-α or deletion of its receptor TNFR1 offers protection from lethal liver damage (27, 28). Interestingly, in the present study, neither LPS/D-GalN-iduced TNF-α nor LPS/D-GalN-iduced TNFR1 expression on hepatocytes were significantly changed by C14-Tri-LAN-Gly pretreatment. Moreover, C14-Tri-LAN-Gly could prevent TNF-α/D-GalN-induced liver injury. Therefore, C14-Tri-LAN-Gly may prevent LPS/D-GalN-induced liver injury by increasing potentially anti-apoptotic genes in hepatocytes.

A20, also known as tumor necrosis factor α-induced protein 3 (TNFAIP3), is essential for the termination of NF-κB signaling in response to TNF and microbial products such as LPS and muramyl dipeptide, but also negatively regulates TNF-induced apoptosis (29). A20 has been shown to be upregulated in hepatocytes by pro-inflammatory stimuli and contribute to liver regeneration after partial hepatectomy and acute toxic hepatitis through combined anti-apoptotic, anti-inflammatory, and pro-proliferative functions (30, 31). In our study, A20 expression in the liver of LPS/D-GalN mice was markedly increased by C14-Tri-LAN-Gly and knockdown of A20 restored liver damage. In addition, C14-Tri-LAN-Gly injection alone also induced a significant increase of A20 expression in hepatocytes, protecting mice from subsequent apoptotic liver damage. Thus, our findings unveil a new role for NOD1 in anti-apoptosis through up-regulation of A20 expression.

A previous study has shown that hepatocytes express high levels of NOD1(17). However, our *in vitro* data showed that treatment of primary hepatocytes with C14-Tri-LAN-Gly could not directly upregulate A20 expression. Instead, we found that TNF-α produced by kupffer cells induced A20 upregulation in hepatocytes following C14-Tri-LAN-Gly treatment. When kupffer cells were depleted or TNF-α was deficient, C14-Tri-LAN-Gly-induced A20 upregulation was impaired. In inflammatory conditions, TNF-α has both a beneficial and a deleterious effect on hepatocytes (32). TNF-α is a proximal mediator of hepatotoxicity in several models of hepatitis and liver damage including LPS/D-GalN (23). Meanwhile, low doses of TNF-α has also been shown to prevent hepatocellular apoptosis and liver damage in inflammatory as well as in ischemia/reperfusion-induced liver injury (33, 34). Our present study also confirmed that TNF-α may play a protective role in liver injury by inducing A20 upregulation in hepatocytes.

Although we and others have used clodronate liposomes to deplete kupffer cells (18), it should be noted that clodronate liposomes are potent in depleting many phagocytic cells including macrophages, circulating monocytes and dendritic cells (DCs). NOD1 is not only expressed in kupffer cells but also in liver sinusoidal endothelial cells (LSECs) and DCs (16, 35), So, our study cannot exclude the possibility that C14-Tri-LAN-Gly also acts on LSECs or DCs or other cells to induce proinflammatory cytokine secretion, which in turn may affect A20 expression in hepatocytes. In addition, apart from TNF-α, IL-1β has also been implicated in upregulating the expression of A20 (29). So, new experiments are needed to puzzle out the full mechanism by which the NOD1 agonist induce A20 upregulation in hepatocytes.

In summary, the present study showed that NOD1 agonist administration ameliorated liver injury and the high lethality induced by either LPS/D-GalN or TNF-α/D-GalN. NOD1 agonist prevented TNF-α-induced hepatocyte apoptosis through the upregulation of A20 expression. NOD1 agonist induced A20 upregulation by Kupffer cell- and TNF-α-dependent mechanisms. The present study also suggests NOD1 as a therapeutic target for liver diseases.

## Data Availability Statement

The original contributions presented in the study are included in the article/supplementary material, further inquiries can be directed to the corresponding author/s.

## Ethics Statement

The animal study was reviewed and approved by Institutional Animal Care and Use Committee of Chongqing medical university.

## Author Contributions

FJ performed the experiments. FJ, ST, and WY designed the experiments and wrote the paper. FJ, FD, PX, SL, XW, PH, HR, ST, and WY analyzed the data. All authors contributed to the article and approved the submitted version.

## Conflict of Interest

The authors declare that the research was conducted in the absence of any commercial or financial relationships that could be construed as a potential conflict of interest.

## Abbreviations

LPS: lipopolysaccharide
D-GalN: D-galactosamine
TNF: tumor necrosis factor
ALF: Acute liver failure
NOD1: Nucleotide oligomerization domain receptor 1
NO: nitric oxide
ALT: Alanine Transminase
TUNEL: terminal deoxynucleotidyl transferase-mediated nick end labeling
MNCs: Liver mononuclear cells
TNFAIP3: tumor necrosis factor α-induced protein 3
LSECs: liver sinusoidal endothelial cells.
