# Supplementary material for: NOD1 Agonist Protects Against Lipopolysaccharide and D-Galactosamine-Induced Fatal Hepatitis Through the Upregulation of A20 Expression in Hepatocytes
Source: Front Immunol. 2021 Mar 4;12:603192. doi: 10.3389/fimmu.2021.603192 (PMC7969647; doi:10.3389/fimmu.2021.603192)
Supplement: Supplementary file 1 [file Data_Sheet_1.docx]

**[Supplementary](javascript:;)**[**materials**](javascript:;)

**NOD1 agonist protects against lipopolysaccharide and D-galactosamine-induced fatal hepatitis through the upregulation of A20 expression in hepatocytes**

Fang Jia, Fuxue Deng, Pan Xu, Shiying Li, Xuefu Wang, Peng Hu, Hong Ren, Shiwen Tong, Wenwei Yin


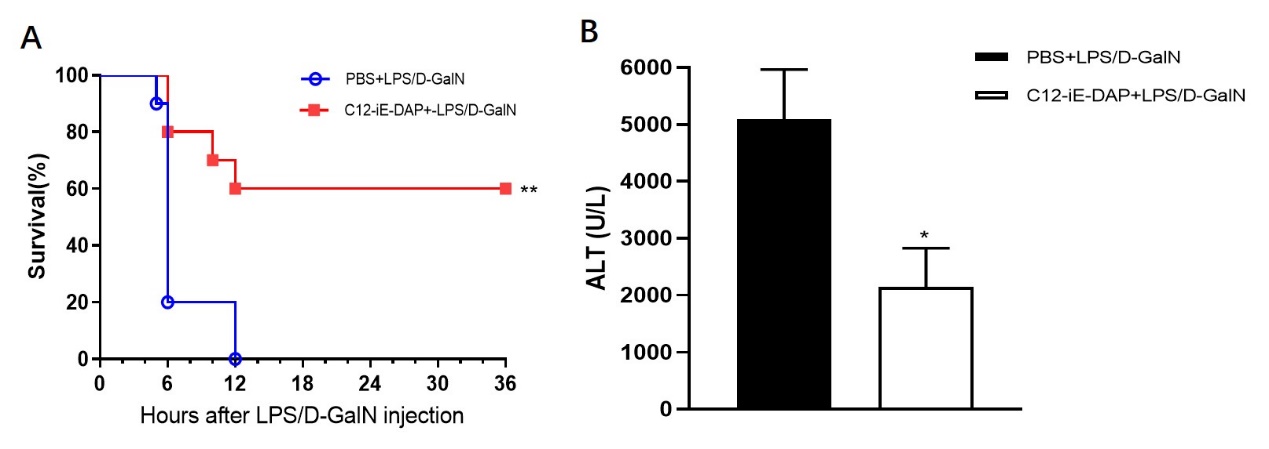


**Supplementary Figure1. C12-iE-DAP protects mice from LPS/D-GalN-induced liver injury.**

Mice were i.p. injected with C12-iE-DAP (10ug/mouse) for 6h. Subsequently, acute liver injury was induced by application of LPS (7.5μg/kg)/D-GalN (500mg/kg). Mice were injected with the same volume of PBS as the vehicle control. (A) Survival curves of LPS/D-GalN mice treated with C12-iE-DAP (n=10). Survival rates were analyzed using the log-rank test. (B) Serum levels of ALT were measured at 6h after LPS/D-GalN injection. * p <0.05, ** p <0.01


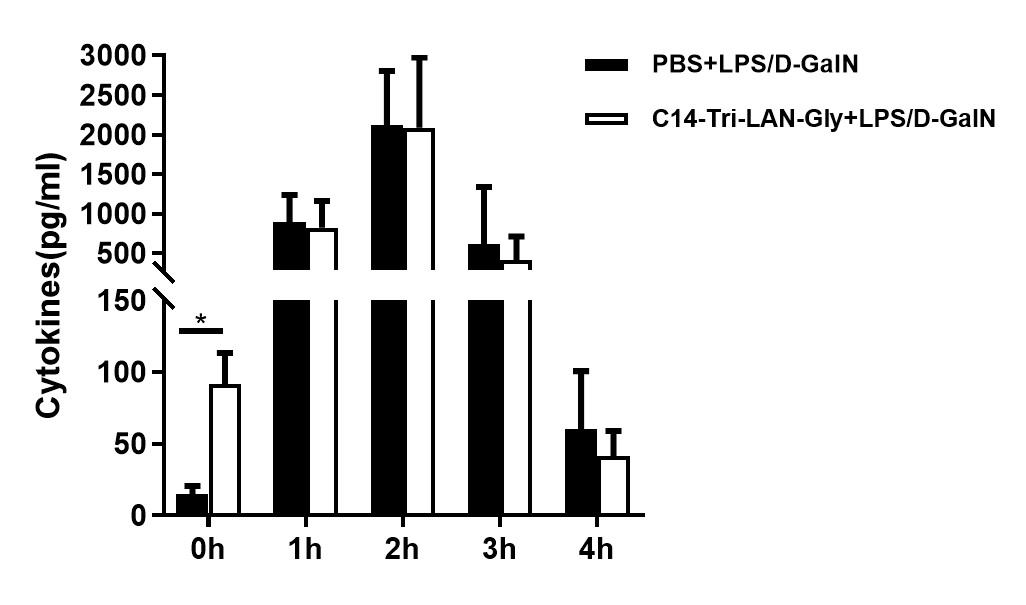


**Supplementary Figure2. C14-Tri-LAN-Gly does not influence TNF-α production.**

Serum TNF-α levels were measured before or after LPS/D-GalN challenge at the indicated times.


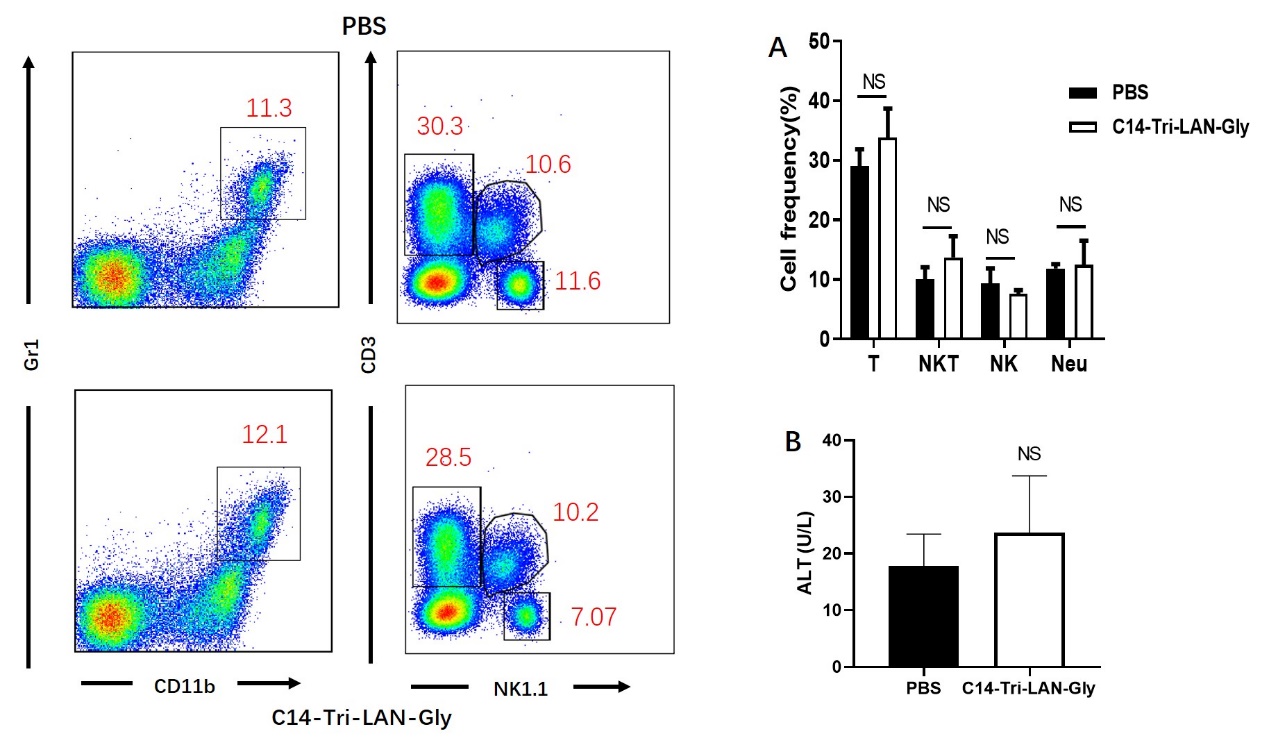


**Supplementary Figure3. C14-Tri-LAN-Gly treatment alone does not affect hepatic leukocyte infiltration.**

(A) Mice were i.p. injected with C14-Tri-LAN-Gly for 6 h, hepatic mononuclear cells were isolated and then marked with specific antibodies. The percentages of hepatic lymphocytes (NK, NKT and T cells) and neutrophils were detected by flow cytometry. (B) Serum level of ALT were measured 6h after C14-Tri-LAN-Gly or PBS challenge.
